# Heart rate variability changes in adolescents following surgical correction of aortic coarctation: Persistent autonomic alterations

**DOI:** 10.14814/phy2.70769

**Published:** 2026-02-06

**Authors:** O. V. Shevaldova, A. V. Kovaleva, A. Yu. Zavarina, E. N. Likhomanova, E. N. Panova, O. B. Obryvchenko

**Affiliations:** ^1^ A.N. Bakulev Center for Cardiovascular Surgery of the Russian Ministry of Health Moscow Russia; ^2^ Federal Research Center for Innovator and Emerging Biomedical and Pharmaceutical Technologies (Anokhin Research Institute of Normal Physiology) Moscow Russia

**Keywords:** adaptation, adolescents, aortic coarctation, autonomic regulation, congenital heart disease, heart rate variability, nonlinear analysis, rehabilitation

## Abstract

Heart rate variability (HRV) is a sensitive marker of autonomic regulation. This study examined adolescents in the long‐term postoperative period after early surgical correction of aortic coarctation (CoA) compared with age‐matched healthy peers. Seventy adolescents (35 CoA, 35 controls; 12–17 years) underwent 5‐min resting ECG and respiratory monitoring. HRV was analyzed using time domain, frequency domain, and nonlinear methods; respiratory rate was included as a covariate in ANCOVA models. Adolescents with repaired CoA showed lower time domain indices (SDNN, RMSSD, pNN50; all *p* < 0.01), reduced total spectral power (*p* = 0.002), and higher VLF (*p* < 0.001). Group differences in SDNN, RMSSD, and the LF/HF remained significant after adjustment for respiratory rate, indicating that autonomic alterations were not explained by breathing patterns. Nonlinear analysis revealed reduced Poincaré plot parameters (SD1, SD2; *p* < 0.01) and higher fractal scaling (DFA Alpha2; *p* < 0.001) in the CoA group, whereas entropy measures and DFA Alpha1 did not differ. These findings demonstrate persistent and selective alterations in autonomic regulation during adolescence despite anatomically successful repair. The coexistence of altered and preserved HRV features suggests domain specific reorganization rather than uniform loss of complexity. Nonlinear HRV indices may improve long term monitoring and help guide individualized rehabilitation.

## 1 INTRODUCTION

Heart rate variability (HRV) is a widely used noninvasive measure of autonomic nervous system function and cardiovascular adaptability, with decreased HRV consistently reported across diverse cardiovascular and psychological conditions (Alieva et al., 2013; Elstad et al., 2018; Ernst, 2017; Schneider & Schwerdtfeger, 2020; Shaffer & Ginsberg, 2017; Sigrist et al., 2021; Thayer et al., 2012). Time‐ and frequency‐domain indices remain standard tools for evaluating autonomic modulation (Reyes del Paso et al., 2013; Tarvainen et al., 2014; Task Force of the European Society of Cardiology and the North American Society of Pacing and Electrophysiology, 1996; Thomas et al., 2019), while nonlinear methods provide complementary information on the complexity and organization of cardiac rhythm regulation (Alexandrov et al., 2017; Assoumou et al., 2010; Bakhchina et al., 2018, 2024; Guzzetti et al., 2005; Ryan et al., 2011).

Aortic coarctation (CoA) accounts for 5%–8% of congenital heart defects (Hoffman & Kaplan, 2002) and causes significant hemodynamic disturbances requiring early surgical repair (Ottaviani & Buja, 2022; Suradi & Hijazi, 2015). Despite anatomically successful correction, adolescents and adults with repaired CoA may continue to exhibit increased aortic stiffness, residual hypertension, or altered baroreflex control (Kenny et al., 2011; Massin & von Bernuth, 1998; Millar et al., 2013; Polson et al., 2006; Ryan et al., 2011). Given the close interplay between cardiovascular structure, hemodynamics, and autonomic regulation, HRV has become an important tool for assessing long‐term physiological consequences of CoA repair.

Existing studies, however, provide inconsistent evidence regarding postoperative autonomic function. Some report reduced HRV and impaired baroreflex sensitivity in children with repaired CoA (Millar et al., 2013), whereas others describe partial or complete normalization several years after early surgery (Igler et al., 1981; Kenny et al., 2009, 2011). These discrepancies may reflect differences in patient age, timing of repair, postoperative duration, and analytical approach. Importantly, most previous research relied primarily on conventional HRV metrics, whereas nonlinear indices, capable of capturing subtle regulatory disturbances, have rarely been examined in this population, despite their growing relevance in physiological and clinical contexts (Fedotova, 2023; Ksela et al., 2009; Lapkin et al., 2012).

Adolescence is a developmental stage marked by rapid maturation of autonomic regulation (Goulopoulou et al., 2010; Pankova, 2008), making it a critical period for detecting persistent abnormalities that may not be apparent earlier in life. Moreover, autonomic alterations have been linked to psychological well‐being and neurocognitive outcomes (Kemp et al., 2010; Koenig et al., 2014; Kovacs et al., 2022), which are particularly relevant for congenital heart disease populations (Kovacs et al., 2022; Toyofuku et al., 2024).

Therefore, the present study aimed to compare HRV in adolescents in the long‐term postoperative period after early surgical correction of CoA with that of healthy peers, using a comprehensive analytical approach that includes time‐domain, frequency‐domain, and nonlinear HRV measures. We hypothesized that despite successful anatomical repair, adolescents with CoA would exhibit persistent alterations in autonomic cardiovascular regulation.

## 2 MATERIALS AND METHODS

The study included 70 adolescents aged 12–17 years. Of these, 35 participants had undergone radical surgical correction for aortic coarctation and were in the long‐term postoperative period (CoA group), receiving rehabilitation at the A.N. Bakulev Center for Cardiovascular Surgery, Moscow, Russia. The remaining 35 participants were healthy adolescents from Bryansk City Lyceum #1, Russia (Normal group).

The majority of children from the CoA group (71%) underwent surgical correction before 1 year of age. The CoA group did not include patients with diagnosed hypertension or those taking medications on a regular basis, as confirmed by a cardiologist. Demographic and anthropometric characteristics of both groups are presented in Tables 1 and 2. Groups were well‐matched for age, sex distribution, and anthropometric parameters, with no statistically significant differences observed between the CoA and Normal groups.

**TABLE 1:** Demographic characteristics of study participants.

	Group		*p* Value
	CoA	Normal	
*N*, persons	35	35	
Age, y (median [25th–75th quartiles])	15.0 [14.0–16.0]	15.0 [14.0–16.0]	0.801^a^
Age range, y	12–17	12–17	
Sex, *n* (%)			
Female	15 (42.9%)	17 (48.6%)	0.81^b^
Male	20 (57.1%)	18 (51.4%)	

*Note*: Data are presented as median [25th–75th quartiles], numbers (%).

^a^ Mann–Whitney *U* test.

^b^ χ^2^‐test.

**TABLE 2:** Anthropometric characteristics of study participants.

	Group		*p* Value^a^
	CoA	Normal	
Height, cm	170 [160.5–176]	168 [163–173]	0.689
Weight, kg	57 [48.5–68.5]	59 [54.5–66.5]	0.634
BMI^b^, kg/m^2^	20.34 [18.035–23.0]	21.41 [19.785–22.96]	0.181

*Note*: Data are presented as median [25th–75th quartiles].

^a^ Mann–Whitney *U* test.

^b^ Body Mass Index.

All participants underwent a 5‐min electrocardiogram (ECG) and pneumogram recording in a sitting position to assess HRV. ECG signals were obtained using a single‐lead configuration with electrodes placed on the wrists. Respiratory activity was recorded simultaneously with the ECG using a respiratory belt sensor (pneumogram).

The respiratory belt was positioned around the abdomen at the level of the umbilicus to predominantly capture abdominal (diaphragmatic) breathing movements.

All recordings were performed under standardized conditions to minimize environmental and physiological variability. Each participant was seated comfortably in a chair with back support in a quiet room with controlled temperature (22°C–24°C) and minimal external stimuli. Before the start of the recording, a 5‐min acclimatization period was provided to allow stabilization of heart rate, blood pressure, and spontaneous respiration.

Participants were instructed to avoid vigorous physical activity, caffeine, and emotional stress for at least 2 h before the assessment. All measurements were performed between 09:00 and 12:00 to reduce circadian influences on HRV.

During the ECG and pneumogram recording, participants were asked to sit still with their hands resting on their thighs, breathe spontaneously without talking, and avoid any movements that could introduce artifacts or influence autonomic tone. For the healthy comparison group, recordings were conducted in a dedicated medical room at school, following the same environmental and procedural standards as in the clinical setting.

To ensure data quality, recordings were visually inspected for artifacts, arrhythmias, and signs of progressive heart rate decline or nonstationary behavior. Traces exhibiting monotonic slowing of heart rate or nonstationary behavior were excluded in accordance with Kubios HRV Standard 3.5.0 software (Tarvainen et al., 2014) analysis recommendations.

RR intervals were extracted from the ECG signal and analyzed using the same software to calculate HRV indices using three analytical approaches: time domain, frequency domain (spectral), and nonlinear analysis. The list of calculated indexes of HRV is presented in Table 3.

**TABLE 3:** Description of HRV parameters assessed in the study.

Index	Definition
Time domain measures	
SDNN, ms	Standard deviation of normal RR‐intervals
RMSSD, ms	Square root of the mean square of the differences of consecutive RR‐intervals
pNN50, %	Percentage of consecutive RR‐intervals differing by more than 50 ms.
Frequency domain measures	
VLF, %	Very low‐frequency component of the heart rate spectrum
LF, %	Low‐frequency component of the heart rate spectrum
HF, %	High‐frequency component of the heart rate spectrum
LF/HF	Low‐ to high‐frequency components ratio
Total power, ms^2^	Total power of the heart rate spectrum
Nonlinear measures	
SD1, ms	Poincaré plot width, dispersion of point projections on a straight line perpendicular to the line of identity
SD2, ms	Poincaré plot length, dispersion of point projections on the line of identity
SD2/SD1	SD2 to SD1 ratio
ApEn	Approximate entropy, an index of heart rate complexity and irregularity
SampEn	Sample entropy, index of heart rate complexity and irregularity
Alpha 1	DFA^a^ short‐term fractal scaling exponent
Alpha 2	DFA long‐term fractal scaling exponent

^a^ Detrended fluctuation analysis.

Simultaneously with ECG acquisition, respiratory rate was recorded using a thoracic belt sensor, allowing assessment of spontaneous breathing during the 5‐min resting measurement. No paced breathing was used. Respiratory frequency (breaths per minute) was extracted from the respiratory waveform and analyzed to account for potential influences on high‐frequency HRV components.

To assess whether between‐group differences in spectral HRV indices were influenced by respiratory rate, we conducted analysis of covariance (ANCOVA) with group (CoA vs. Normal) as a fixed factor and respiratory rate (breaths/min) as a covariate. Respiratory activity exerts its strongest effects on vagally mediated short‐term variability and high‐frequency spectral components (Hayano et al., 1994; Solinsky et al., 2022); thus, the assessment of respiratory influence was focused on HF, LF/HF, SDNN, and RMSSD for which separate ANCOVA models were constructed. Nonlinear measures and longer‐range variability indices are not substantially driven by instantaneous breathing patterns and therefore do not necessitate adjustment for respiratory rate.

The nonparametric Mann–Whitney *U* test was used to compare HRV indices between the CoA and control groups, as the data did not follow normal distribution for all parameters (Kolmogorov–Smirnov test). Data are presented as median [25th–75th quartiles]. All statistical analyses were performed using Statistica 12 software (StatSoft Inc., Tulsa, OK, USA). A *p* value < 0.05 was considered statistically significant.

The study was conducted in full accordance with the Declaration of Helsinki (1975, revised in 2008 and updated in 2024 for studies initiated before 2025), and was approved by the Ethics Committee of the P.K. Anokhin Research Institute of Normal Physiology (Approval No. 18/1, 15 February 2023).

Prior to participation, parents (legal representatives) of all adolescents received written and verbal explanations of the study goals, procedures, potential benefits, and possible risks. The study involved only noninvasive, minimal‐risk procedures (5‐min resting ECG and pneumogram), and parents were informed that participation would not affect medical care or school activities. Only after ensuring full understanding, parents provided written informed consent for their child's participation and for the use of anonymized data for research and publication.

Although participants were under the legal age of consent, all adolescents (12–17 years old) were capable of understanding the procedures and aims of the study, and each provided written assent prior to participation. The research team confirmed that adolescents understood that participation was voluntary and that they could withdraw at any time without consequences.

In accordance with the Declaration of Helsinki, individuals not legally capable of providing informed consent are considered particularly vulnerable and must be included only when the research involves minimal risk and burden or is expected to provide individual benefit. Both criteria were met in our study for both groups:

1. CoA group: Participants underwent only noninvasive ECG and pneumogram recording, which are part of their routine cardiological follow‐up and therefore involved no additional burden beyond standard care;
2. Normal group: Participation also involved only minimal‐risk, noninvasive ECG and pneumogram recording performed in a quiet room at school.

No medications were administered, and no interventions, stress tests, or procedures beyond standard noninvasive assessment were used. All data were anonymized prior to analysis.

## 3 RESULTS

HRV parameters were compared between adolescents with repaired CoA and healthy controls. The results are summarized in Table 4.

**TABLE 4:** HRV indices and respiratory rate in adolescents after aortic coarctation repair compared to healthy controls.

Index	CoA group	Normal group	*p* Value^a^
Time domain			
**SDNN, ms**	27.35 [22.61–39.85]	43.18 [34.64–51.57]	**0.0059**
**RMSSD, ms**	26.27 [17.24–38.59]	39.36 [31.81–49.41]	**0.0041**
RR, ms	735.35 [653.65–769.28]	749.53 [693.04–822.13]	0.2263
HR, bpm	81.59 [78.0–91.8]	79.76 [72.67–86.06]	0.1481
**pNN50, %**	5.46 [1.05–14.01]	18.06 [8.49–33.1]	**0.0006**
Frequency domain			
**VLF, %**	6.22 [3.3–9.72]	2.48 [0.99–3.11]	**<0.001**
LF, %	52.81 [33.24–68.12]	45.24 [30.8–59.7]	0.4109
HF, %	37.85 [21.14–58.93]	49.82 [35.3–67.24]	0.1239
LF/HF	1.45 [0.59–3.08]	0.86 [0.45–1.45]	0.0778
**Total power, ms** ^**2**^	657.25 [447.38–1561.17]	1734.77 [1003.19–2228.47]	**0.0023**
Nonlinear			
**SD1, ms**	18.62 [12.22–27.35]	27.89 [22.54–35.02]	**0.0041**
**SD2, ms**	34.82 [29.56–50.68]	55.11 [41.4–64.43]	**0.0031**
SD2/SD1	2.05 [1.72–2.32]	2.04 [1.63–2.21]	0.481
ApEn	1.01 [0.93–1.06]	0.96 [0.93–1.02]	0.1792
SampEn	1.8 [1.52–1.88]	1.65 [1.49–1.83]	0.5259
Alpha 1	1.04 [0.898–1.31]	1.10 [0.856–1.23]	0.448
**Alpha 2**	0.45 [0.32–0.57]	0.25 [0.2–0.36)	**<0.001**
Respiratory rate			
**Respiratory rate (breaths per minute)**	18.7 [17.5–20.0]	15.9 [14.9–17.0]	**<0.001**

*Note*: Data are presented as median [25th–75th quartiles]; Statistically significant differences between groups are highlighted in bold.

^a^ Mann–Whitney *U* test.

As shown in Table 4, the CoA group exhibited markedly reduced overall heart rate variability, with significantly lower SDNN, RMSSD, and pNN50 values compared with controls, indicating diminished parasympathetic modulation. Mean RR interval and heart rate did not differ significantly between groups. In the frequency domain, total spectral power was substantially lower in the CoA group, while the relative contribution of the VLF component was significantly higher. No significant differences were found in the LF or HF components or in the LF/HF ratio, although the latter showed a trend toward higher values in adolescents with repaired CoA.

Because respiratory rate differed significantly between groups (*p* < 0.001), we conducted ANCOVA to determine whether between‐group differences in HRV indices were influenced by breathing frequency. Respiratory rate was entered as a covariate, and group (CoA vs. control) as a fixed factor. In the spectral domain, respiratory rate was not a significant covariate for HF power or LF/HF ratio (Table 5). HF power remained non‐different between groups after adjustment for respiratory rate. In contrast, LF/HF showed a significant group difference after adjustment, reflecting a clearer separation between groups when variability related to breathing frequency was accounted for.

**TABLE 5:** ANCOVA results for HRV indices with respiratory rate as a covariate.

HRV index	Effect of group (CoA vs. control)	*p* Value (group)	Effect of respiration	*p* Value (respiration)
HF, %	ns	0.534	ns	0.236
LF/HF	**↑ in CoA**	**0.026**	ns	0.829
SDNN, ms	**↓ in CoA**	**0.0007**	ns	0.460
RMSSD, ms	**↓ in CoA**	**0.0003**	Weak negative effect	0.043

*Note*: ANCOVA models included Group (fixed factor) and Respiratory rate (covariate). Arrows indicate direction of effect relative to control. Statistically significant differences between groups are highlighted in bold.

Abbreviation: ns, nonsignificant.

In the time domain, SDNN and RMSSD remained significantly lower in the CoA group after adjustment for respiratory rate. Respiratory rate was not a significant covariate for SDNN and showed only a modest negative association with RMSSD (Table 5).

Nonlinear indices (Table 4) further highlighted differences in cardiac rhythm organization. Both short‐term (SD1) and long‐term (SD2) components of HRV represented by Poincaré plot parameters were significantly lower in the CoA group. The SD2/SD1 ratio did not differ significantly between groups.

**FIGURE 1:**
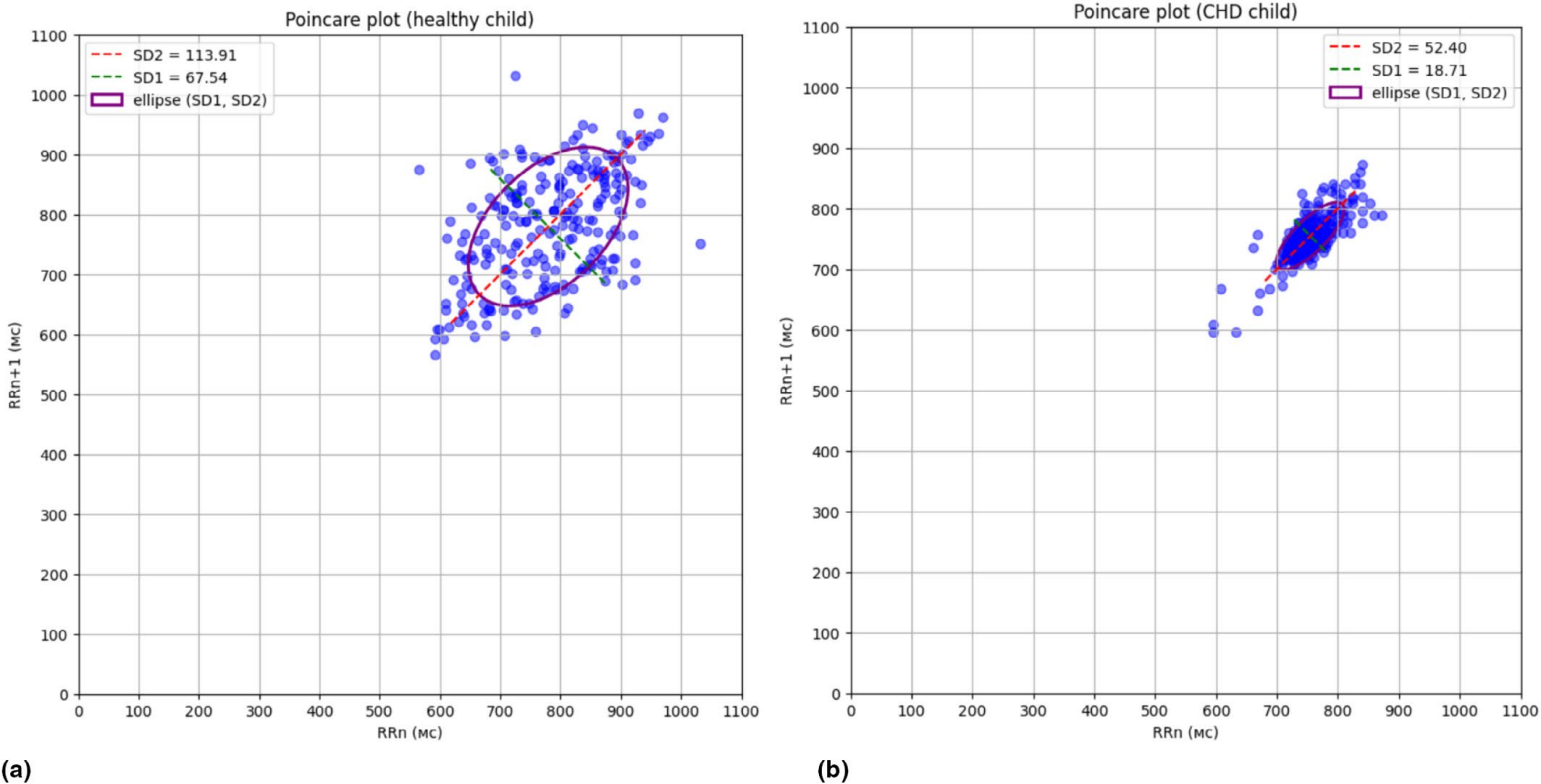
Poincaré plots of resting state RR – intervals of healthy adolescent (a) and adolescent after radical surgical correction for CoA (b).

Figure 1 illustrates representative Poincaré plots from a healthy adolescent (a) and an adolescent with CoA (b). The plots visually demonstrate the reduced SD1 and SD2 values in the CoA group, reflecting decreased beat‐to‐beat variability. Despite comparable mean heart rates in these individual examples (80 bpm in the CoA adolescent vs. 77 bpm in the healthy adolescent), the CoA patient shows a more compact, concentrated pattern of points, indicating reduced overall variability in both short‐term and long‐term components.

Comparison of fractal scaling properties showed different patterns for short‐ and long‐range dynamics. The short‐term exponent (DFA Alpha 1) did not differ between groups. In contrast, the long‐term exponent (DFA Alpha 2) was higher in adolescents with repaired CoA. Given that DFA Alpha 2 is known to be less reliable when derived from short ECG recordings, this difference should be interpreted with caution and may reflect methodological rather than physiological factors. Entropy‐based measures (ApEn and SampEn) also showed no between‐group differences.

## 4 DISCUSSION

Children in the long‐term period after congenital heart disease surgery undergo comprehensive examination by cardiologists and rehabilitation specialists, including ECG monitoring. However, medical specialists typically evaluate these findings from a clinical perspective focused on cardiovascular abnormalities, with less attention paid to autonomic and central nervous system regulatory influences on the functional state of these patients. Psychological rehabilitation elements, including functional state correction using biofeedback training (including cardiorespiratory and HRV‐BFB protocols), are increasingly incorporated into rehabilitation programs for congenital heart disease (CHD) patients. Therefore, it was necessary to investigate HRV and autonomic regulation processes in these children to develop effective, safe, and individualized protocols.

Our study demonstrates significant differences in cardiac autonomic regulation between adolescents who underwent aortic coarctation repair in early childhood and their healthy peers. On one hand, these differences align with expectations for patients with a history of significant cardiovascular intervention. On the other hand, the literature provides contradictory evidence regarding long‐term autonomic regulation in such patients, with some studies noting persistent hemodynamic and heart rate regulatory disturbances (Millar et al., 2013; Suarez‐Roca et al., 2021; Suradi & Hijazi, 2015), while others report normalization (Igler et al., 1981; Kenny et al., 2009).

Kenny et al. (2009) demonstrated that autonomic function indices measured by noninvasive methods in patients with early correction of CoA normalized 5 years after surgery based on baroreflex sensitivity assessment. These results are consistent with data from an animal model showing normalization of baroreflex sensitivity in dogs after CoA correction (Igler et al., 1981). Our findings, however, suggest persistent alterations in cardiac autonomic regulation during adolescence in the long‐term postoperative period based on HRV parameters.

Our analysis revealed differences between healthy adolescents and CHD patients across all three types of heart rate analysis, with the most pronounced differences in time domain parameters. SDNN, which reflects total HRV by including all oscillatory components affecting variability during the observation period, was significantly lower in the CoA group. This reduction may indicate lower adaptive capabilities of the cardiovascular system in these patients and may be associated with residual hemodynamic disorders or changes in autonomic cardiac control following surgical intervention (Task Force of the European Society of Cardiology and the North American Society of Pacing and Electrophysiology, 1996).

RMSSD, an index of short‐term HRV reflecting predominantly parasympathetic activity (Shaffer & Ginsberg, 2017), was also reduced in the CoA group, suggesting prolonged weakening of vagal control over heart rate. Similarly, lower values of pNN50 in the CoA group confirmed the weakening of vagal control in the long‐term postoperative period (Stein et al., 1994).

Because respiratory frequency influences high‐frequency HRV components and differed between groups, ANCOVA was used to assess whether breathing accounted for the observed autonomic differences. After adjustment, HF power remained comparable between groups, whereas a significant difference in the LF/HF ratio emerged, indicating that respiration‐related variability had partially masked this effect in the unadjusted analysis. In contrast, time‐domain indices (SDNN and RMSSD) remained markedly lower in the CoA group after adjustment, and respiratory rate showed no meaningful influence on these measures. These results indicate that the reduced time‐domain HRV indices primarily reflect intrinsic autonomic alterations rather than differences in breathing patterns.

Spectral analysis of heart rate revealed significant differences between groups only for total spectral power and VLF power. The lower total power in the CoA group represents reduced overall activity of regulatory influences on heart rate, potentially indicating a prolonged decrease in the adaptive potential of the cardiovascular system (Billman, 2011).

The VLF component of the heart rate spectrum may reflect various extracardiac regulatory influences, including higher brain regions and hormonal/metabolic processes (Assoumou et al., 2010; Guzzetti et al., 2005; Penaz, 1978; Ryan et al., 2011; Taylor et al., 1998). Interestingly, the CoA group exhibited significantly higher VLF values compared to controls. This finding may reflect activation of additional adaptive mechanisms (neural and hormonal) of autonomic regulation in these adolescents. Previous research has demonstrated a relationship between VLF power and cognitive load. For example, Usui and Nishida (Usui & Nishida, 2017) showed that following performance of the Stroop test as a stress model, VLF was the only HRV spectral index that reflected prolonged recovery within 2 h of mental load.

It is noteworthy that other spectral indices (LF, HF, and LF/HF ratio), which are commonly used in CHD studies, showed no significant differences between our comparison groups. This highlights the importance of employing a comprehensive approach to HRV analysis that includes nonlinear methods.

Nonlinear indices, although rarely used in clinical studies, proved to be highly informative when comparing resting state ECG between adolescents with CHD and healthy controls. The presence of significant nonlinearities in the heart regulation system determines the chaotic nature of heart rate, where each subsequent RR interval differs from others and is unique (Braun et al., 1998).

In our study, both Poincaré plot parameters (SD1 and SD2) were significantly lower in the CoA group compared to the control group. SD1 reflects short‐term variability primarily associated with respiratory sinus arrhythmia and parasympathetic activity, while SD2 reflects long‐term variability more influenced by the sympathetic nervous system (Guzik et al., 2007). The reduction in both parameters suggests comprehensive alterations in autonomic cardiac regulation affecting both rapid vagal responses and slower sympathetic influences. We found only one conference proceeding addressing Poincaré plot analysis in children with and without congenital heart disease, specifically in infants (Smith et al., 2009), but it does not directly address our findings in adolescents with repaired CoA.

Previous research in pediatric populations with congenital heart disease has generally documented reduced HRV and signs of autonomic dysregulation in the long‐term postoperative period, and nonlinear heart rate variability analysis has been proposed as a sensitive tool for detecting such alterations (Akiya et al., 2025; Aletti et al., 2012). For example, children and adolescents with complex CHD after Tetralogy of Fallot or Fontan repair demonstrated significant changes in nonlinear HRV indices compared with healthy peers, suggesting altered autonomic control after surgery (Akiya et al., 2025). Standard measures of HRV have also been shown to be lower in children after congenital heart surgery compared with controls (Hami & Corcia, 2017). These alterations have typically been interpreted as indicators of decreased autonomic flexibility related to chronic sympathetic predominance and long‐standing postoperative remodeling of cardiac regulatory mechanisms. In contrast to this established pattern, adolescents with CoA in our study did not demonstrate reductions in some nonlinear HRV metrics. DFA Alpha 1 was preserved, DFA Alpha 2 tended to be higher in the CoA group (although this index should be interpreted cautiously due to the short 5‐min duration of recordings), and both approximate entropy (ApEn) and sample entropy (SampEn) showed no significant between‐group differences, with slightly higher median values in the CoA cohort.

Entropy‐based indices are commonly interpreted as markers of the irregularity, unpredictability, and complexity of cardiac dynamics. Earlier work, including studies by Bakhchina et al. (2024) and Bakhchina et al. (2018), demonstrates that higher ApEn and SampEn values reflect more differentiated, less stereotyped physiological patterns. Within this context, the absence of decreased entropy and the preservation of DFA Alpha 1 in adolescents after CoA repair suggest that long‐term postoperative autonomic dynamics may not follow the conventional trajectory of reduced complexity reported in prior CHD research. Instead, these findings may reflect reorganization or compensatory adaptation of autonomic regulation during adolescence, resulting in nonlinear characteristics that diverge from the expected deficit‐based profile.

This pattern may be understood in light of the conceptual framework proposed by Alexandrov et al. (2017), according to which individuals in pathological conditions develop new forms of adaptation to altered environments. During long‐term recovery after surgery, adolescents with CHD acquire new behavioral and physiological strategies, leading to a more differentiated interaction with the environment. Such adaptive reorganization may result not in a simple “loss of complexity,” as expected from classical HRV literature, but in the emergence of alternative, compensatory modes of complexity.

Our findings align with contemporary theoretical perspectives that view physiological and behavioral regulation as dynamic, adaptive, and capable of reorganizing under chronic or developmentally altered conditions. Models such as allostasis (McEwen & Wingfield, 2003; Sterling, 2012) and the neurovisceral integration framework (Thayer & Lane, 2000) emphasize that variability in physiological signals—including heart rate fractal properties—reflects the organism's capacity to develop flexible and differentiated regulatory strategies rather than simply reverting to a normative pattern. Similar principles are embedded in fractal and network physiology (Goldberger et al., 2002; Ivanov et al., 2016; Peng et al., 1995), where complexity is understood as an emergent property that may increase, decrease, or transform depending on the adaptive demands imposed by long‐term alterations in health or environment.

These theoretical views are further supported by empirical evidence showing that chronic conditions, including congenital heart disease, can promote not only reductions but also qualitative shifts in nonlinear HRV organization. Our results, characterized by preserved entropy measures, DFA Alpha 1 and altered DFA Alpha 2 in adolescents in the long‐term postoperative period after surgical correction of coarctation, are consistent with this broader understanding of adaptive reconfiguration. This integrated perspective provides a plausible framework for interpreting nonlinear HRV features in surgically corrected CHD populations as indicators of adaptive physiological restructuring during adolescence.

## 5 LIMITATIONS

Several limitations of this study should be acknowledged. First, the cross‐sectional design precludes causal inference regarding the origins of the observed autonomic differences. Longitudinal studies following children from the early postoperative period into adolescence are required to determine whether these HRV alterations represent persistent consequences of early surgical intervention, adaptive responses developing over time, or individual variability unrelated to surgery.

Second, although participants with repaired CoA were not receiving regular cardiovascular medications at the time of examination according to cardiologist evaluation, we cannot fully exclude the potential influence of prior pharmacological treatment or intermittent medication use on autonomic regulation. Additionally, lifestyle factors such as habitual physical activity, sleep patterns, diet, and psychosocial stress were not quantified in this study. These variables are known to affect HRV and may contribute to inter‐individual variability in both the clinical and control groups.

Third, although respiratory rate was recorded and statistically adjusted for in ANCOVA models, respiration itself was not controlled or paced. Participants breathed spontaneously, which implies that variability in respiratory depth, pattern, and thoracoabdominal contributions may have introduced residual variance, particularly in indices sensitive to respiratory mechanics. Thus, statistical adjustment for respiratory rate cannot fully account for the physiological effects of unconstrained breathing.

Fourth, the nonlinear index DFA Alpha 2 was derived from 5‐min ECG segments. Short‐term recordings provide reliable information primarily about the short‐range scaling exponent (Alpha 1), whereas estimation of Alpha 2 typically requires longer time series to capture true long‐term fractal correlations. Therefore, the Alpha 2 values reported here should be interpreted cautiously, as they may not fully reflect the long‐range fractal organization of heart rate dynamics.

Taken together, these limitations indicate that the present findings should be interpreted cautiously and highlight the need for future longitudinal, multimodal studies incorporating controlled assessment of respiratory parameters, detailed quantification of lifestyle behaviors, and comprehensive clinical history.

## 6 CONCLUSIONS

This study demonstrates that adolescents in the long‐term period after successful anatomical repair of aortic coarctation exhibit persistent alterations in autonomic cardiovascular regulation. These alterations were most evident in reduced time‐domain HRV indices (SDNN, RMSSD, and pNN50), reflecting diminished parasympathetic activity and reduced overall autonomic modulation. Importantly, these between‐group differences remained robust after statistical adjustment for respiratory rate, indicating that the autonomic disturbances identified here cannot be explained solely by differences in breathing frequency.

Beyond conventional HRV indices, nonlinear measures provided additional and clinically informative insights into cardiac rhythm organization. Reduced Poincaré plot parameters (SD1 and SD2) and altered long‐range fractal scaling (DFA Alpha2) revealed regulatory modifications not captured by spectral metrics alone.

Equally important, several nonlinear characteristics did not differ between groups. Both entropy‐based indices (ApEn and SampEn) and the short‐range fractal exponent (DFA Alpha 1) were preserved in adolescents with repaired CoA. The coexistence of changed (SD1, SD2, and DFA Alpha 2) and preserved (ApEn, SampEn, and DFA Alpha 1) nonlinear features suggests a selective reorganization rather than a uniform reduction of cardiac rhythm complexity in the long‐term postoperative period.

Together, these findings indicate that autonomic dysregulation persists into adolescence despite early surgical correction of CoA, a developmental stage characterized by ongoing maturation of cardiovascular and neurophysiological systems. This underscores the need for continued long‐term surveillance and supports the potential utility of HRV‐based assessments, including nonlinear analyses, for identifying individuals who may benefit from targeted rehabilitation or autonomic modulation strategies.

Future studies incorporating longitudinal designs, extended ECG recordings, baroreflex sensitivity evaluation, and multimodal physiological assessments are warranted to clarify the mechanisms underlying these persistent autonomic alterations and to determine the clinical applicability of nonlinear HRV metrics in adolescents with congenital heart disease.

## AUTHOR CONTRIBUTIONS

O.V. Shevaldova and A.V. Kovaleva drafted manuscript; A.Yu. Zavarina, E.N. Likhomanova, E.N. Panova, and O.B. Obryvchenko edited and revised manuscript; A.V. Kovaleva, O.V. Shevaldova, and A.Yu. Zavarina approved final version of manuscript.

## FUNDING INFORMATION

This research was supported by the Federal Research Center for Innovator and Emerging Biomedical and Pharmaceutical Technologies (research topic #122040500027‐7: “Investigation of system physiological mechanisms of psychoemotional stress and pain reactions” (FGFW‐2022‐0001)) and A.N. Bakulev Center for cardiovascular surgery of the Russian Ministry of Health (topic of applied scientific research #123020300024‐9: “Development of individual rehabilitation programs in children after correction of congenital heart disease at early and remote stages of rehabilitation period (DVHB‐2023‐0018)”).

## ETHICS STATEMENT

The study was conducted in full accordance with the Declaration of Helsinki (1975, revised in 2008 and updated in 2024 for studies initiated before 2025), and was approved by the Ethics Committee of the P.K. Anokhin Research Institute of Normal Physiology (Approval No. 18/1, 15 February 2023).

## Data Availability

The raw data supporting the conclusions of this article are publicly available in the Zenodo repository at https://doi.org/10.5281/zenodo.17896121. The dataset includes anonymized heart rate variability measurements and demographic characteristics for all study participants.

## References

[phy270769-bib-0001] Akiya, A. , Takahashi, K. , Iwahara, K. , Akatsuka, Y. , Sato, H. , Sato, K. , Kago, H. , Shigemitsu, S. , Fukunaga, H. , Akimoto, K. , & Kishiro, M. (2025). Impact of nonlinear heart rate variability in postoperative complex congenital heart disease: Insights from repaired tetralogy of Fallot and Fontan palliation. Pediatric Cardiology, 46, 1–9. 10.1007/s00246-025-04020-2 41003713

[phy270769-bib-0002] Aletti, F. , Ferrario, M. , de Jesus, T. B. , Stirbulov, R. , Silva, A. B. , Cerutti, S. , & Sampaio, L. M. (2012). Heart rate variability in children with cyanotic and acyanotic congenital heart disease: Analysis by spectral and non linear indices. Annual International Conference of the IEEE Engineering in Medicine and Biology Society, 2012, 4189–4192. 10.1109/EMBC.2012.6346890 23366851

[phy270769-bib-0003] Alexandrov, Y. I. , Svarnik, O. E. , Znamenskaya, I. I. , Arutyunova, K. R. , Kolbeneva, M. G. , Krylov, A. K. , & Bulava, A. I. (2017). Stress, illness and learning as the conditions of regression. Voprosy Psikhologii, 4, 87.

[phy270769-bib-0004] Alieva, A. M. , Golukhova, E. Z. , & Pinchuk, T. V. (2013). Heart rate variability rhythm in chronic heart failure (review). The Russian Archives of Internal Medicine, 6, 47–52. 10.20514/2226-6704-2013-0-6-47-52

[phy270769-bib-0005] Assoumou, H. G. , Pichot, V. , Barthelemy, J. C. , Dauphinot, V. , Celle, S. , Gosse, P. , Assoumou, H. G. N. , Kossovsky, M. , Gaspoz, J. M. , & Roche, F. (2010). Metabolic syndrome and short‐term and long‐term heart rate variability in elderly free of clinical cardiovascular disease: The PROOF study. Rejuvenation Research, 13, 653–663. 10.1089/rej.2010.1019 20818933

[phy270769-bib-0006] Bakhchina, A. V. , Demidovsky, A. V. , & Alexandrov, Y. I. (2018). Correspondence between the heart rate complexity and system characteristics of performed behavior. Psikhologicheskii Zhurnal, 39(5), 46–58. 10.31857/S020595920000834-3

[phy270769-bib-0007] Bakhchina, A. V. , Sozinova, I. S. , & Alexandrov, Y. I. (2024). Dynamics of neurovisceral interactions in individual and phylogenetic development: Analysis of heart rate variability. Neuroscience and Behavioral Physiology, 54, 1242–1255. 10.1007/s11055-024-01722-7

[phy270769-bib-0008] Billman, G. E. (2011). Heart rate variability—A historical perspective. Frontiers in Physiology, 2, 86. 10.3389/fphys.2011.00086 22144961 PMC3225923

[phy270769-bib-0009] Braun, C. , Kowallik, P. , Freking, A. , Hadeler, D. , Kniffki, K. D. , & Meesmann, M. (1998). Demonstration of nonlinear components in heart rate variability of healthy persons. American Journal of Physiology—Heart and Circulatory Physiology, 275(5), H1577–H1584. 10.1152/ajpheart.1998.275.5.h1577 9815063

[phy270769-bib-0010] Elstad, M. , O'Callaghan, E. L. , Smith, A. J. , Ben‐Tal, A. , & Ramchandra, R. (2018). Cardiorespiratory interactions in humans and animals: Rhythms for life. American Journal of Physiology—Heart and Circulatory Physiology, 315, H6–H17. 10.1152/ajpheart.00701.2017 29522373

[phy270769-bib-0011] Ernst, G. (2017). Heart‐rate variability—More than heart beats? Frontiers in Public Health, 5, 240. 10.3389/fpubh.2017.00240 28955705 PMC5600971

[phy270769-bib-0012] Fedotova, E. V. (2023). Prospects for using indicators of non‐linear analysis of heart rate variability as markers of the functional state of the athlete's body when performing training and testing loads. Teoriia i Praktika Fizicheskoĭ Kul'tury, 2023(10), 26–29.

[phy270769-bib-0013] Goldberger, A. L. , Peng, C.‐K. , & Lipsitz, L. A. (2002). What is physiologic complexity and how does it change with aging and disease? Neurobiology of Aging, 23, 23–26. 11755014 10.1016/s0197-4580(01)00266-4

[phy270769-bib-0014] Goulopoulou, S. , Fernhall, B. , & Kanaley, J. A. (2010). Developmental changes in hemodynamic responses and cardiovagal modulation during isometric handgrip exercise. International Journal of Pediatrics, 2010, 153780. 10.1155/2010/153780 20862202 PMC2938431

[phy270769-bib-0015] Guzik, P. , Piskorski, J. , Krauze, T. , Schneider, R. , Wesseling, K. H. , Wykrȩtowicz, A. , & Wysocki, H. (2007). Correlations between the Poincaré plot and conventional heart rate variability parameters assessed during paced breathing. Journal of Physiological Sciences, 57(1), 63–71. 10.2170/physiolsci.RP005506 17266795

[phy270769-bib-0016] Guzzetti, S. , la Rovere, M. T. , Pinna, G. D. , Maestri, R. , Borroni, E. , Porta, A. , Mortara, A. , & Malliani, A. (2005). Different spectral components of 24 h heart rate variability are related to different modes of death in chronic heart failure. European Heart Journal, 26(4), 357–362. 10.1093/eurheartj/ehi067 15618038

[phy270769-bib-0017] Hami, K. , & Corcia, M. C. G. (2017). Heart rate variability modifications after surgery for congenital heart disease in young patients. Evidence‐Based Medicine Practice, 3, 113. 10.4172/2471-9919.1000113

[phy270769-bib-0018] Hayano, J. , Mukai, S. , Sakakibara, M. , Okada, A. , Takata, K. , & Fujinami, T. (1994). Effects of respiratory interval on vagal modulation of heart rate. American Journal of Physiology, 267(1 Pt 2), H33–H40. 10.1152/ajpheart.1994.267.1.H33 7914066

[phy270769-bib-0019] Hoffman, J. I. E. , & Kaplan, S. (2002). The incidence of congenital heart disease. Journal of the American College of Cardiology, 39, 1890–1900. 10.1016/s0735-1097(02)01886-7 12084585

[phy270769-bib-0020] Igler, F. O. , Boerboom, L. E. , Werner, P. H. , Donegan, J. H. , Zuperku, E. J. , Bonchek, L. I. , & Kampine, J. P. (1981). Coarctation of the aorta and baroreceptor resetting. A study of carotid baroreceptor stimulus‐response characteristics before and after surgical repair in the dog. Circulation Research, 48(3), 365–371. 10.1161/01.res.48.3.365 7460210

[phy270769-bib-0021] Ivanov, P. C. , Liu, K.‐K. L. , & Bartsch, R. P. (2016). Focus on the emerging new fields of network physiology and network medicine. New Journal of Physics, 18, 100201. 30881198 10.1088/1367-2630/18/10/100201 PMC6415921

[phy270769-bib-0022] Kemp, A. H. , Quintana, D. S. , Gray, M. A. , Felmingham, K. L. , Brown, K. , & Gatt, J. M. (2010). Impact of depression and antidepressant treatment on heart rate variability: A review and meta‐analysis. Biological Psychiatry, 67(11), 1067–1074. 10.1016/j.biopsych.2009.12.012 20138254

[phy270769-bib-0023] Kenny, D. , Polson, J. W. , Martin, R. P. , Caputo, M. , Wilson, D. G. , Cockcroft, J. R. , Paton, J. F. , & Wolf, A. R. (2011). Relationship of aortic pulse wave velocity and baroreceptor reflex sensitivity to blood pressure control in patients with repaired coarctation of the aorta. American Heart Journal, 162(2), 398–404. 10.1016/j.ahj.2011.05.020 21835303

[phy270769-bib-0024] Kenny, D. , Polson, J. W. , Martin, R. P. , Paton, J. F. R. , & Wolf, A. R. (2009). Normalization of autonomic function in children with coarctation of the aorta after surgical correction in infancy. Hypertension, 54, e21–e22. 10.1161/hypertensionaha.109.136192 19667246

[phy270769-bib-0025] Koenig, J. , Jarczok, M. N. , Ellis, R. J. , Hillecke, T. K. , & Thayer, J. F. (2014). Heart rate variability and experimentally induced pain in healthy adults: A systematic review. European Journal of Pain (United Kingdom), 18, 301–314. 10.1002/ejp.1749 23922336

[phy270769-bib-0026] Kovacs, A. H. , Brouillette, J. , Ibeziako, P. , Jackson, J. L. , Kasparian, N. A. , Kim, Y. Y. , Livecchi, T. , Sillman, C. , Kochilas, L. K. , & American Heart Association Council on Lifelong Congenital Heart Disease and Heart Health in the Young; and Stroke Council . (2022). Psychological outcomes and interventions for individuals with congenital heart disease: A scientific Statement from the American Heart Association. Circulation. Cardiovascular Quality and Outcomes, 15(8), e000110. 10.1161/HCQ.0000000000000110 35862009

[phy270769-bib-0027] Ksela, J. , Suwalski, P. , Kalisnik, J. M. , Avbelj, V. , Suwalski, G. , & Gersak, B. (2009). Assessment of nonlinear heart rate dynamics after beating‐heart revascularization. The Heart Surgery Forum, 12(1), E10–E16. 10.1532/HSF98.20081104 19233759

[phy270769-bib-0028] Lapkin, M. M. , Vikhrov, S. P. , Alpatov, A. V. , & Mitrofanova, M. Y. (2012). Fractal‐fluctuation analysis of heart rate nonlinear components to parameterize the functional condition. I.P. Pavlov Russian Medical Biological Herald, 20(2), 96. 10.17816/pavlovj2012296-106

[phy270769-bib-0029] Massin, M. , & von Bernuth, G. (1998). Clinical and haemodynamic correlates of heart rate variability in children with congenital heart disease. European Journal of Pediatrics, 157(12), 967–971. 10.1007/s004310050979 9877033

[phy270769-bib-0030] McEwen, B. S. , & Wingfield, J. C. (2003). The concept of allostasis in biology and biomedicine. Hormones and Behavior, 43, 2–15. 12614627 10.1016/s0018-506x(02)00024-7

[phy270769-bib-0031] Millar, P. J. , Proudfoot, N. A. , Dillenburg, R. F. , & MacDonald, M. J. (2013). Reduced heart rate variability and baroreflex sensitivity in normotensive children with repaired coarctation of the aorta. International Journal of Cardiology, 168(1), 587–588. 10.1016/j.ijcard.2013.01.248 23453876

[phy270769-bib-0032] Ottaviani, G. , & Buja, L. M. (2022). Congenital heart disease: Pathology, natural history, and interventions. In Cardiovascular Pathology. Elsevier. 10.1016/B978-0-12-822224-9.00011-6

[phy270769-bib-0033] Pankova, N. B. (2008). Functional development of the vegetative regulation of the cardiovascular system in human ontogenesis. Rossiiskii Fiziologicheskii Zhurnal Imeni I.M. Sechenova/Rossiiskaia Akademiia Nauk, 94(3), 267–275. 18507155

[phy270769-bib-0034] Penaz, J. (1978). Mayer waves: History and methodology. Automedica, 2(3), 135–141.

[phy270769-bib-0035] Peng, C.‐K. , Havlin, S. , Stanley, H. E. , & Goldberger, A. L. (1995). Quantification of scaling exponents and crossover phenomena in nonstationary heartbeat time series. Chaos, 5, 82–87. 11538314 10.1063/1.166141

[phy270769-bib-0036] Polson, J. W. , McCallion, N. , Waki, H. , Thorne, G. , Tooley, M. A. , Paton, J. F. R. , & Wolf, A. R. (2006). Evidence for cardiovascular autonomic dysfunction in neonates with coarctation of the aorta. Circulation, 113(24), 2844–2850. 10.1161/CIRCULATIONAHA.105.594986 16769911

[phy270769-bib-0037] Reyes del Paso, G. A. , Langewitz, W. , Mulder, L. J. , van Roon, A. , & Duschek, S. (2013). The utility of low frequency heart rate variability as an index of sympathetic cardiac tone: A review with emphasis on a reanalysis of previous studies. Psychophysiology, 50, 477–487. 10.1111/psyp.12027 23445494

[phy270769-bib-0038] Ryan, M. L. , Ogilvie, M. P. , Pereira, B. M. T. , Gomez‐Rodriguez, J. C. , Manning, R. J. , Vargas, P. A. , Duncan, R. C. , & Proctor, K. G. (2011). Heart rate variability is an independent predictor of morbidity and mortality in hemodynamically stable trauma patients. Journal of Trauma: Injury, Infection & Critical Care, 70(6), 1371–1380. 10.1097/TA.0b013e31821858e6 21817974

[phy270769-bib-0039] Schneider, M. , & Schwerdtfeger, A. (2020). Autonomic dysfunction in posttraumatic stress disorder indexed by heart rate variability: A meta‐analysis. Psychological Medicine, 50, 1937–1948. 10.1017/S003329172000207X 32854795 PMC7525781

[phy270769-bib-0040] Shaffer, F. , & Ginsberg, J. P. (2017). An overview of heart rate variability metrics and norms. Frontiers in Public Health, 5, 258. 10.3389/fpubh.2017.00258 29034226 PMC5624990

[phy270769-bib-0041] Sigrist, C. , Reichl, C. , Schmidt, S. J. , Brunner, R. , Kaess, M. , & Koenig, J. (2021). Cardiac autonomic functioning and clinical outcome in adolescent borderline personality disorder over two years. Progress in Neuro‐Psychopharmacology & Biological Psychiatry, 111, 110336. 10.1016/j.pnpbp.2021.110336 33915219

[phy270769-bib-0042] Smith, R. L. , Wathen, E. R. , Abaci, P. C. , von Bergen, N. H. , Law, I. H. , Dick, M. D. , Connor, C. , & Dove, E. L. (2009). Analyzing heart rate variability in infants using non‐linear Poincaré techniques. Computers in Cardiology, 36, 673–676.

[phy270769-bib-0043] Solinsky, R. , Schleifer, G. D. , Draghici, A. E. , Hamner, J. W. , & Taylor, J. A. (2022). Methodologic implications for rehabilitation research: Differences in heart rate variability introduced by respiration. PM & R: The Journal of Injury, Function, and Rehabilitation, 14(12), 1483–1489. 10.1002/pmrj.12770 PMC9309192 35077032

[phy270769-bib-0044] Stein, P. K. , Bosner, M. S. , Kleiger, R. E. , & Conger, B. M. (1994). Heart rate variability: A measure of cardiac autonomic tone. American Heart Journal, 127(5), 1376–1381. 10.1016/0002-8703(94)90059-0 8172068

[phy270769-bib-0045] Sterling, P. (2012). Allostasis: A model of physiological regulation. Physiology & Behavior, 106, 5–15. 21684297 10.1016/j.physbeh.2011.06.004

[phy270769-bib-0046] Suarez‐Roca, H. , Mamoun, N. , Sigurdson, M. I. , & Maixner, W. (2021). Baroreceptor modulation of the cardiovascular system, pain, consciousness, and cognition. Comprehensive Physiology, 11(2), 1373–1423. 10.1002/cphy.c190038 33577130 PMC8480547

[phy270769-bib-0047] Suradi, H. , & Hijazi, Z. M. (2015). Current management of coarctation of the aorta. Global Cardiology Science and Practice, 2015, 44. 10.5339/gcsp.2015.44 26779519 PMC4710863

[phy270769-bib-0048] Tarvainen, M. P. , Niskanen, J. P. , Lipponen, J. A. , Ranta‐aho, P. O. , & Karjalainen, P. A. (2014). Kubios HRV—Heart rate variability analysis software. Computer Methods and Programs in Biomedicine, 113(1), 210–220. 10.1016/j.cmpb.2013.07.024 24054542

[phy270769-bib-0049] Task Force of the European Society of Cardiology and the North American Society of Pacing and Electrophysiology . (1996). Heart rate variability: Standards of measurement, physiological interpretation and clinical use. Circulation, 93, 1043–1065. 10.1161/01.cir.93.5.1043 8598068

[phy270769-bib-0050] Taylor, J. A. , Carr, D. L. , Myers, C. W. , & Eckberg, D. L. (1998). Mechanisms underlying very‐low‐frequency RR‐interval oscillations in humans. Circulation, 98(6), 547–555. 10.1161/01.cir.98.6.547 9714112

[phy270769-bib-0051] Thayer, J. F. , Åhs, F. , Fredrikson, M. , Sollers, J. J. , & Wager, T. D. (2012). A meta‐analysis of heart rate variability and neuroimaging studies: Implications for heart rate variability as a marker of stress and health. Neuroscience and Biobehavioral Reviews, 36, 747–756. 10.1016/j.neubiorev.2011.11.009 22178086

[phy270769-bib-0052] Thayer, J. F. , & Lane, R. D. (2000). A model of neurovisceral integration in emotion regulation and dysregulation. Journal of Affective Disorders, 61, 201–216. 11163422 10.1016/s0165-0327(00)00338-4

[phy270769-bib-0053] Thomas, B. L. , Claassen, N. , Becker, P. , & Viljoen, M. (2019). Validity of commonly used heart rate variability markers of autonomic nervous system function. Neuropsychobiology, 78, 14–26. 10.1159/000495519 30721903

[phy270769-bib-0054] Toyofuku, A. , Ehrler, M. , Naef, N. , Schmid, A. S. , Kretschmar, O. , Latal, B. , & O'Gorman Tuura, R. (2024). Heart rate variability and cognitive functions in adolescents with complex congenital heart disease. Pediatric Research, 97, 1103–1113. 10.1038/s41390-024-03432-9 39080463 PMC12055568

[phy270769-bib-0056] Usui, H. , & Nishida, Y. (2017). The very low‐frequency band of heart rate variability represents the slow recovery component after a mental stress task. PLoS One, 12(8), e0182611. 10.1371/journal.pone.0182611 28806776 PMC5555691

